# Dynamics of infectious disease spread between transportation hubs and surrounding communities

**DOI:** 10.1016/j.idm.2026.05.009

**Published:** 2026-06-06

**Authors:** Rahele Mosleh, Mina Shafadeh, Bushra Majeed, Julien Arino, Abbas Ghasemi, Edward W. Thommes, Ali Asgary, Herman Hui, Jianhong Wu

**Affiliations:** a Laboratory for Industrial and Applied Mathematics (LIAM), York University, Toronto, Ontario, Canada; b Department of Mathematics, University of Manitoba, Winnipeg, Manitoba, Canada; c Mechanical and Industrial Engineering Department, Toronto Metropolitan University, Toronto, Ontario, Canada; d Department of Mathematics and Statistics, University of Guelph, Guelph, Ontario, Canada; e New Products and Innovation, Sanofi, Toronto, Ontario, Canada; f Disaster and Emergency Management Unit, School of Administrative Studies, Faculty of Liberal Arts and Professional Studies, and Advanced Disaster, Emergency and Rapid Response Simulation (ADERSIM), York University, Toronto, Ontario, Canada; g Research and Analytics Department, Toronto Transit Commission (TTC), Toronto, Ontario, Canada; h Centre of Excellence in Artificial Intelligence for Public Health Advancement (AIPHA), York University, Toronto, Ontario, Canada

## Abstract

Urban transit systems, particularly those in major metropolitan areas, are becoming increasingly interconnected, making it essential to better understand passenger mobility and its implications for the spread of infectious diseases. Respiratory infectious diseases, including COVID-19 (as a case study), can spread rapidly in densely populated urban environments, particularly within public transportation systems. This study investigates the dynamics of infection spread at a subway station, and its surrounding community, focusing on both short-term and long-term transmission. Using two deterministic ordinary differential equation (ODE) models, we simulate disease transmission over a single business day and examine how daily encounters impact infection numbers over the subsequent three months. The short-term model captures localized interactions between transit passengers and community residents, while the long-term model evaluates the cumulative impact of these encounters on community infections. Parameter estimation was performed using ridership data and the least squares method. Results suggest that mobility-related factors, particularly inflow and outflow rates, have a greater impact on controlling disease spread than transmission rates in both short- and long-term dynamics. Reducing inflow eases congestion and lowers encounters in the hub but increases encounters in the community, whereas reducing outflow increases crowding in the hub while decreasing encounters in the community. Joint reductions in inflow and outflow decrease encounters in both settings. In the long term, changes in transmission rates have only a limited effect on peak infections, while reducing outflow notably decreases infections and reducing inflow slightly increases community infections. Overall, simultaneously reducing inflow and outflow is the most effective strategy for limiting encounters in the short term and infections in the long term.

## 1 Introduction

**Fig. 1:**
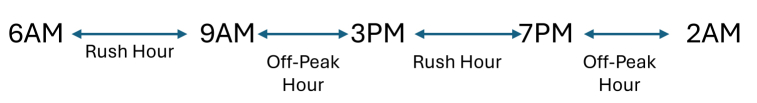
Timeline of Finch Station operations.

Respiratory infectious diseases, such as SARS, influenza, and COVID-19, are highly transmissible in densely populated environments, often leading to outbreaks and epidemics. Public transportation, particularly subway systems in large cities, plays a crucial role in the daily commute for millions of people (Wen et al., 2020). During epidemics, subway usage significantly increases the risk of disease transmission due to high population density, frequent usage, and continuous passenger flow. In confined spaces like subway trains and stations, the close proximity of passengers heightens the transmission risk. Passengers who become infected can further spread pathogens to their families and others via other contact mixing. Infected individuals, often unaware of their illness, may expose fellow passengers to the virus. Contributing factors include crowded platforms, limited ventilation, and frequent contact with contaminated surfaces, all of which facilitate the spread of infectious agents (Goscé & Johansson, 2018). Subway systems, which connect densely populated urban areas, enable the rapid dissemination of diseases within and between cities. Understanding the dynamics of disease transmission within these transit networks is vital for implementing effective public health interventions to prevent the spread. As has been shown in previous studies (Buonanno et al., 2020; Liu et al., 2017; Setti et al., 2020), diseases can easily spread without direct physical contact in transportation hubs. Airborne diseases, such as SARS, influenza, and COVID-19, are primarily transmitted through respiratory emissions from infected individuals, including sneezing, coughing, or speaking. Researchers have employed various mathematical models to address the role of transportation in epidemic spread, investigating factors such as passenger movement, airflow, and surface contamination. Classical compartmental models based on ordinary differential equations (ODEs) are commonly used to study infectious disease epidemics, particularly respiratory diseases. For instance, Hyman and LaForce employed differential equations to model disease spread in cities, validating their findings with real influenza data (Hyman, 2003). Arino and van den Driessche developed a multi-city epidemic model to analyze disease dynamics and identify critical thresholds for spread (Arino & van den Driessche, 2003). Li, Peikun et al. used the SEIR (Susceptible-Exposed-Infected-Recovered) model to assess COVID-19 transmission risk among subway commuters, accounting for factors like disinfection, ventilation, journey duration, transmission capacity, and passenger fluctuations (Li et al., 2022). Various deterministic models have been proposed to study the impact of transport-related infection on disease spread and control between different cities and regions (Qian & Ukkusuri, 2021; Takeuchi & Saito, 2006; Wan et al., 2007; Xu et al., 2013). In addition to these deterministic models, many researchers have developed agent-based models (ABMs) to analyze the impact of transportation on disease transmission. For instance, Cooley, Philip et al. (Cooley et al., 2011) simulated an agent-based model for New York City's five boroughs to investigate the role of subway riders in influenza epidemics, finding that 4 − 5% of flu infections occurred on subways. Guo et al. (Guo et al., 2023) and Li et al. (Li et al., 2022) investigated COVID-19 transmission risk among subway commuters using the SEIR model. Guo and colleagues further employed an individual-based dynamic model of a town's rail system, demonstrating that all residents eventually became infected, with 86.6% of infections attributed to public transport. The study in (Zhou et al., 2023) models COVID-19 transmission in subway systems using passenger flow data and an SIR framework, demonstrating that combined disinfection and off-peak travel can reduce infections in busy stations by approximately 50%. Hong et al. assessed the COVID-19 transmission risk across various transport modes, linking proximity and exposure time to the identification of high-risk settings and guide targeted prevention measures (Ye et al., 2025). In this work, we develop two deterministic ordinary differential equation (ODE) models to capture station-level disease transmission over short-term and long-term time scales. The short-term model provides a high-resolution view of localized transmission within a single business day, offering insights that are often overlooked in broader frameworks. This localized perspective constitutes a key novelty of the study, as it enables the evaluation of immediate intervention strategies through the optimization of transmission and mobility rates. The long-term model examines how individuals encountered during a single business day influence infection dynamics over the subsequent three months. Another central novelty lies in explicitly modeling transmission between a transportation hub and its surrounding community and assessing the long-term impact of one-day encounters on future infection levels. For the short-term dynamics, model parameters are estimated across different time intervals, represented in Fig. 1, using the least squares method. The primary objective of this study is to quantify the number of individuals encountered in both the community and the hub during short-term dynamics and to assess how community encounters influence the number of infected individuals in the community over long-term dynamics. Notably, Our findings indicate that mobility factors, particularly inflow and outflow rates, have a greater impact on limiting disease spread than transmission parameters in both short- and long-term settings. Specifically, reducing inflow alleviates congestion and decreases encounters within the hub, but increases encounters in the surrounding community. In contrast, decreasing outflow raises crowd density and encounters in the hub while reducing interactions in the community, thereby indirectly increasing transmission risk within the hub. Furthermore, simultaneous reductions in both inflow and outflow decrease encounters in both settings. From a long-term perspective, modifying either community or hub transmission rates produces only marginal reductions in the peak number of infections. By comparison, lowering inflow during a single business day slightly increases long-term community infections, whereas reducing outflow results in a more pronounced decrease in infections, consistent with the short-term behavior. Overall, concurrent reductions in inflow and outflow lead to fewer infected individuals in the community and emerge as the most effective strategy for reducing community encounters in the short term and community infections in the long term.

## 2 Background information

Finch Station, located in the heart of Toronto's North York area in Canada, serves as a key transit hub with eight entrances, offering extensive connectivity and accessibility. Its large parking area, with over 3000 spaces, connects to the lower concourse via tunnels, where passengers can wait for their transportation (Finch station, 2024). Additionally, there is a dedicated lane near the GO bus terminal, positioning Finch Station as a central hub for various transportation needs in the region. To investigate the mechanism of outbreak dissemination within an urban transit hub, we model as a case study the COVID-19 outbreak at Finch Station, part of the Toronto Transit Commission (TTC) subway system, using a deterministic SEIR (Susceptible, Encountered, Infectious, Recovered) model. In this model, individuals in the encountered compartment represent those who have been encountered to the infection by contact with infectious individuals and are in the very early stage of exposure. The population is divided into two distinct clusters: the hub (subway station) and the surrounding community, allowing us to capture interactions between commuters at the station and residents in the neighboring area. This division enables us to assess the interaction between encountered individuals within the subway station and its impact on the surrounding community's exposure. Our key objective is to quantify the number of individuals encountered to the virus within the hub and evaluate how this exposure affects the number of encountered individuals in the surrounding community over a single business day. To capture the dynamics of this exposure, we examine passenger mobility and disease spread across four time intervals: morning rush hour, midday off-peak hours, afternoon rush hour, and evening off-peak hours. These intervals cover the station's operational hours, running from 6 a.m. to 2 a.m. the following day, reflecting varying passenger density and mobility patterns throughout the day. Using the deterministic SEIR model, we track the progression of the outbreak during these time intervals, incorporating the inflow and outflow rates of passengers at each period. This approach allows for a detailed understanding of how commuter movement within the hub influences the overall transmission risk in the community.

Fig. 1 shows the time division interval of ridership at Finch Station over a single business day, based on TTC website data (TTC, 2024). The intervals are defined as follows: 6 a.m. to 9 a.m. as the morning rush hour, 9 a.m. to 3 p.m. as the midday off-peak hours, 3 p.m. to 7 p.m. as the afternoon rush hours, and 7 p.m. to 2 a.m. the following day as the evening off-peak hours. Based on actual data provided by the TTC's data analysis section, including ridership statistics from fare gates, buses, and subway services over a single day in November 2022, we conducted a comprehensive analysis of these datasets. This analysis involved examining passenger flow patterns, identifying peak travel times, and understanding the distribution of commuters across various transit modes. These insights allowed us to estimate key parameters such as inflow and outflow rates at different times of the day, which are essential for modeling the spread of infectious diseases within the transit system.

**Fig. 2:**
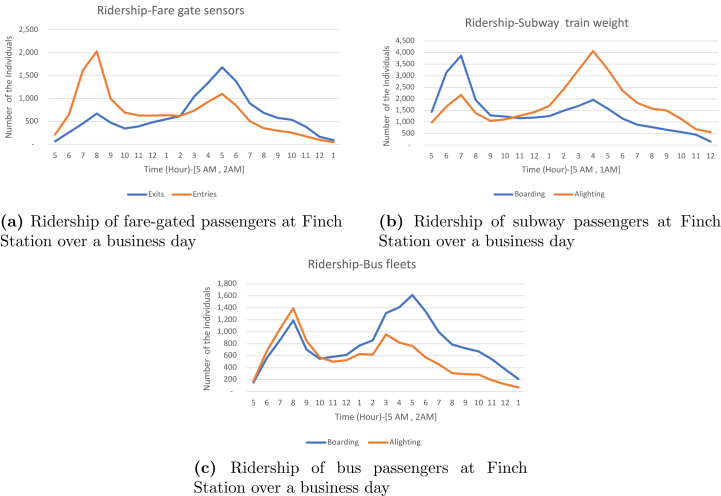
Ridership of passengers at Finch Station over a business day based on the actual data.

Finch Station is an integrated station, meaning the bus terminal is located inside the fare-paid area of the station. There are several methods to inform passenger usage of the station, including fare gate sensor counts to track station walk-ins and walk-outs, subway train weight counts to inform subway boardings and alightings at the platform level, automatic passenger counting (APC) devices on 100% of the bus fleet to track the number of passengers picked up or dropped off at the station. Fig. 2 showcases the ridership of passengers at Finch Station through these three methods over a typical business day. Fig. 2a shows the ridership through the fare gate at Finch Station during a single business day. It tracks two activities: entries (in orange) and exits (in blue) throughout the day. Based on the actual data, from 5 a.m. to 6 a.m., 215 passengers tap on the fare gate and enter the station. The number of passengers entering the station increases during the morning rush hours, reaching its maximum between 8AM and 9 a.m. with 2031 passengers. Afterward, the number of passengers decreases during the midday off-peak hours, from 9 a.m. to 3 p.m. The second rush hour starts at 3 p.m., with the number of passengers reaching its maximum between 5 p.m. and 6 p.m. with 1101 passengers tapping on. Following this, the afternoon off-peak hours begin, with the minimum number of passengers tapping on between 1 a.m. and 2 a.m., totaling 50 persons. The figure shows that in the morning, more passengers enter the station than exit through the fare gate. During the peak of the morning rush hours, the number of passengers exiting the station is one-third of those entering. The number of passengers exiting through the fare gate increases over time, reaching its maximum during the afternoon rush hours between 5 p.m. and 6 p.m. with 1682 passengers, which is greater than the number of passengers tapping on and entering through the fare gate at the same time. Afterward, the number of passengers exiting through the fare gate decreases over time and reaches its minimum between 1 a.m. and 2 a.m., totaling 94 persons. Overall, the total number of passengers entering, 14,077 persons, is greater than those exiting, 13,124 persons, over a single business day in November 2022. As shown in Fig. 2b, the ridership pattern of the subway boarding (in blue) and alighting (in orange) is opposite to that of the fare gate. Boarding peaks at 3862 passengers during the morning rush hour from 8 a.m. to 9 a.m., while alighting also rises, reaching a peak of 2165 passengers, though this peak is significantly lower than that of boarding. During the afternoon rush hour, alighting peaks at 4070 passengers between 5 p.m. and 6 p.m., while boarding increases to 1,985, still significantly lower. This trend indicates that more passengers are exiting the subway than entering it. Overall, the total number of passengers alighting, 35,434 persons, is greater than those boarding, 27,896 persons, over the course of the day. Fig. 2c depicts the ridership pattern of the bus for passengers boarding and alighting. The maximum number of passengers alighting is slightly greater than those boarding during the morning between 8 a.m. and 9 a.m. Throughout the day, the trend shows that passengers alighting from the buses generally falls below those boarding. The maximum number of passengers alighting in the afternoon rush hours, between 5 p.m. and 6 p.m., is almost half of the maximum number of passengers boarding the bus during the same time. Overall, the number of passengers boarding the bus, 16,802 persons, is greater than those alighting, 11,812 persons, by the end of the day.

Remark 2.1 It is worth noting that, within this framework, all values on the time axis represent one-hour intervals. For example, the label 5 corresponds to the time period from 5:00 to 5:59.

**Fig. 3:**
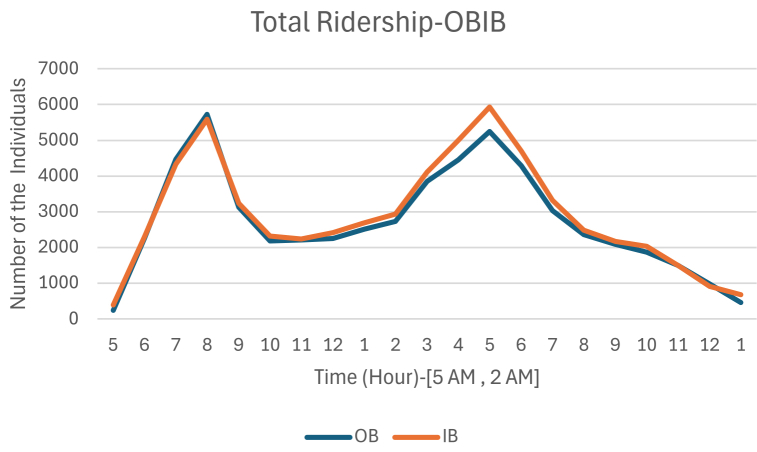
Ridership of total outbound and inbound passengers at Finch Station over a business day.

Fig. 3 illustrates the total ridership patterns at Finch Station over a single business day in November 2022. For the purposes of this analysis, Finch Station is defined as a bounded transit hub. The figure shows the total number of passengers entering the station (depicted in orange) and exiting the station (depicted in blue). Outbound (OB) data represent passengers tapping off at the fare gates as they board subways and buses, while inbound (IB) data represent passengers tapping on at the fare gates as they alight from subways and buses. This visualization allows us to identify key trends in passenger flow throughout the day. The analysis provides important insights into the station's usage patterns and peak travel hours. Because the total number of daily commuters is almost the same whether measured using inbound or outbound counts, this study focuses on the inbound (IB) case. Approximately 61,323 passengers pass through Finch Station each day between 5 a.m. and 2 a.m. the following morning. This information is essential for understanding commuter behavior, optimizing transit operations, and assessing the potential risk of disease transmission within the station.

## 3 Methodology

**Fig. 4:**
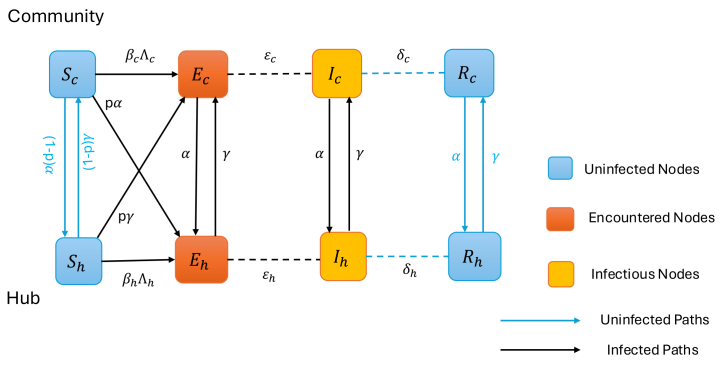
Schematic representation of disease transmission dynamics within a subway station and its surrounding community.

**Fig. 5:**
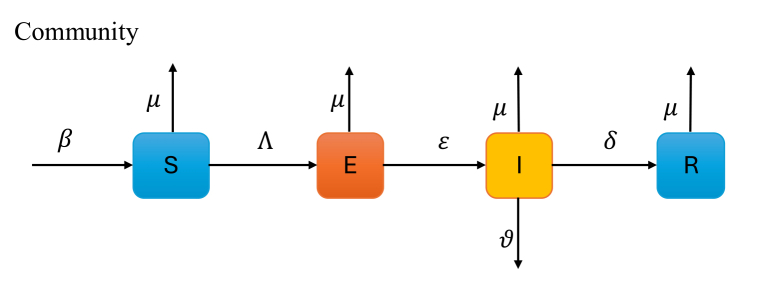
Schematic representation of disease transmission dynamics within a community.

In this study, we focus on Finch Subway Station in North York, Canada, as a case study, along with its surrounding community. We examine the impact of COVID-19 on individuals commuting between the hub and the community and on community residents over a single day. Ridership data from a business day in November 2022 are used to represent the short-term dynamics. We then explore how encounters within the community during this single day affect the number of infected individuals over the subsequent three months, referred to as the long-term dynamics. To achieve this, two systems of equations are employed: one for short-term and one for long-term dynamics. Short-term dynamics are modeled using a deterministic ODE system with coupled SEIR clusters (community and hub), as shown in model 1 and Fig. 4, where the total population remains constant for integrated both clusters over a single day. Long-term dynamics are modeled with a deterministic SEIR-based ODE system (model 4 and Fig. 5), in which the total population of the community is determined by the ratio of birth and natural mortality rates (Equation (10)) in long run. Regarding the short-term dynamics, although ridership data are available, the derived inflow and outflow rates do not perfectly match the model 1 requirements. Therefore, we estimate key parameters, including the hub transmission and mobility rates, using the least squares method. Since operating hours contain critical time intervals (Fig. 1), parameters are estimated separately for morning rush hours, mid-day off-peak hours, afternoon rush hours, and evening off-peak hours, as summarized in Table 2. Using these estimates, we determine the number of encountered individuals in both the community and the hub over a single day and assess the impact of protection measures on infection transmission under short-term dynamics. Finally, we investigate how community encounters during one day influence infection numbers over the following three months, including the long-term effects of short-term protection measures.

**Table 1:** Parameters, descriptions, values, and references.

Parameter	Description	Value	Reference
*β*_*c*_	Rate of the community transmission rate	0.0627 hour^−1^	Majeed et al. (2023)
*ɛ*_*c*_	Rate of the progression from the early latent phase to the infectious phase within the community	0.01041 − 0.0208hour^−1^	(Jansen, 2021; Ogata & Tanaka, 2023; Song et al., 2022)
*ɛ*_*h*_	Rate of the progression from the early latent phase to the infectious phase within the hub	1.041e − 3 − 2.08e − 3hour^−1^	(Jansen, 2021; Ogata & Tanaka, 2023; Song et al., 2022) a
*δ*_*c*_	Recovery rate within the community	4.1e − 3hour^−1^	CDC (2021)
*δ*_*h*_	Recovery rate within the hub	4.1e − 4hour^−1^	(CDC, 2021) a
*ν*	Disease death rate	1.4e − 6day^−1^	Public Health Ontario (2023)
*μ*	Natural mortality rate	2.08e − 5day^−1^	(Government of Ontario & Ministry of Finance, 2025; Public Health Ontario, 2023; Statistics Canada, 2025)
*β*	Birth rate	2.32e − 5day^−1^	Croteau (2024)
*β*_*c*_	Rate of the community transmission rate	1.5054 day^−1^	Majeed et al. (2023)
*ɛ*	Rate of the progression from the early latent phase to the infectious phase within the community	0.25 − 0.5day^−1^	(Jansen, 2021; Ogata & Tanaka, 2023; Song et al., 2022)
*δ*	Recovery rate within the community	1/10day^−1^	CDC (2021)

a Since passengers commute more quickly than the community members within the hub and have shorter stay durations, we assume that the progression rate from the encountered phase to the infectious phase, as well as the recovery rate in the hub, is 1/10 of their respective values in the community.

**Table 2:** Parameters, descriptions, and estimated values.

Parameter	Description	Estimated Value	95% Confidence Interval
$\beta_{h_{rush\text{\_}mor}}$	Transmission rate of the hub in the morning rush-hour time interval	0.8048 hour^−1^	[0.0.79600, 0.80531]
$\beta_{h_{o\mathrm{ff}\text{\_}mid}}$	Transmission rate of the hub in the midday peak-off time interval	0.1138 hour^−1^	[0.11354, 0.114832]
$\beta_{h_{rush\text{\_}aft}}$	Transmission rate of the hub in the afternoon rush-hour time interval	0.7901 hour^−1^	[0.78993, 0.79115]
$\beta_{h_{o\mathrm{ff}\text{\_}eve}}$	Transmission rate of the hub in the evening off-peak time interval	0.3734 hour^−1^	[0.370977, 0.37495]
*α*_*rush*_*mor*_	Inflow rate from the community to the hub in the morning rush-hour time interval	0.78668 hhour^−1^	[0.786600, 0.78811]
*α*_*off*___*mid*_	Inflow rate from the community to the hub in the midday peak-off time interval	0.1336 hour^−1^	[0.13237, 0.13380]
*α*_*rush*_*aft*_	Inflow rate from the community to the hub in the afternoon rush-hour time interval	0.7252 hour^−1^	[0.71853, 0.72566]
*α*_*off*___*eve*_	Inflow rate from the community to the hub in the evening off-peak time interval	0.0633 hour^−1^	[0.06329, 0.06365]
*γ*_*rush*_*mor*_	Outflow rate from the hub to the community in the morning rush-hour time interval	0.5607 hour^−1^	[0.56061, 0.56329]
*γ*_*off*___*mid*_	Outflow rate from the hub to the community in the midday peak-off time interval	0.3226 hour^−1^	[0.32166, 0.32570]
*γ*_*rush*_*aft*_	Outflow rate from the hub to the community in the afternoon rush-hour time interval	0.2982 hour^−1^	[0.29219, 0.29867]
*γ*_*off*___*eve*_	Outflow rate from the hub to the community in the evening off-peak time interval	0.4187 hour^−1^	[0.41796, 0.41893]
$k_{c_{\mathrm{rush}}}$	Saturation number of the community in the rush-hour time interval	0.5479	[0.54353, 0.54822]
$k_{c_{\mathrm{off}}}$	Saturation number of the community in the off-peak hour time interval	0.5095	[0.50943, 0.51165]
$k_{h_{\mathrm{rush}}}$	Saturation number of the hub in the rush-hour time interval	0.01026	[0.01022, 0.01088]
$k_{h_{\mathrm{off}}}$	Saturation number of the hub in the off-peak hour time interval	0.00141	[0.00117, 0.00143]
*N*_*c*_	Population size of the community surrounding Finch Station	7, 040	-
*I*_0*c*_	Initial number of infectees in the community	60	-
*p*	Percentage of individuals transferring infection	0.0003	[3.1810, 3.5093]*e* − 04

## 4 Modeling process

In this section, we employ two systems of deterministic ordinary differential equations (ODEs) to model COVID-19 transmission between Finch Subway Station, a major transportation hub, and its surrounding community. The first system captures short-term dynamics, quantifying how individuals encounter the infection within a single business day. The second system extends the analysis to long-term dynamics, assessing how daily community encounters influence the number of infected individuals in the community over the subsequent three months. These two models allow us to identify key drivers of transmission and mobility rate, evaluate the relative impact of encounters in the hub versus the community, and predict long-term outcomes under specific intervention or mobility scenarios. This approach is particularly useful for informing targeted strategies to reduce infection risk and manage crowding in public transportation settings. Notably, we assume that the initial number of encountered individuals in the community is zero.

### 4.1 Short-term dynamics

We have developed a deterministic compartmental SEIR (Susceptible, Encountered, Infectious, Recovered) model to examine the transmission dynamics of COVID-19 outbreak within Finch Subway Station, considered as the hub, and its surrounding community in the North York region, located in Canada as a case study. In this model, the encountered compartment includes individuals in the very early stage of exposure who have contracted the infection through contact with infectious individuals commuting between the hub and its surrounding community. This framework captures interactions between different population groups, enabling analysis of how the infection spreads both within Finch Station and the adjacent community. By segmenting the population into distinct compartments—passengers within the hub and individuals residing in the community—the model provides a detailed understanding of outbreak progression, the impact of various transmission factors, and the effectiveness of potential interventions. The ODE model, illustrated in Fig. 4, is given below:

(1) $$\begin{array}{ll} {\dot{S}}_{c}\left(t\right) & =-\beta_{c}\Lambda_{c}\left(t\right)S_{c}\left(t\right)-\alpha S_{c}\left(t\right)+\left(1-p\right)\gamma S_{h}\left(t\right)\\ {\dot{E}}_{c}\left(t\right) & =\beta_{c}\Lambda_{c}\left(t\right)S_{c}\left(t\right)-\epsilon_{c}E_{c}\left(t\right)+p\gamma S_{h}\left(t\right)-\alpha E_{c}\left(t\right)+\gamma E_{h}\left(t\right)\\ {\dot{I}}_{c}\left(t\right) & =\epsilon_{c}E_{c}\left(t\right)+\gamma I_{h}\left(t\right)-\alpha I_{c}\left(t\right)-\delta_{c}I_{c}\left(t\right)\\ {\dot{R}}_{c}\left(t\right) & =\delta_{c}I_{c}\left(t\right)-\alpha R_{c}\left(t\right)+\gamma R_{h}\left(t\right)\\ {\dot{S}}_{h}\left(t\right) & =-\beta_{h}\Lambda_{h}\left(t\right)S_{h}\left(t\right)-\gamma S_{h}\left(t\right)+\alpha \left(1-p\right)S_{c}\left(t\right)\\ {\dot{E}}_{h}\left(t\right) & =\beta_{h}\Lambda_{h}\left(t\right)S_{h}\left(t\right)-\epsilon_{h}E_{h}\left(t\right)+p\alpha S_{c}\left(t\right)-\gamma E_{h}\left(t\right)+\alpha E_{c}\left(t\right)\\ {\dot{I}}_{h}\left(t\right) & =\epsilon_{h}E_{h}\left(t\right)-\delta_{h}I_{h}\left(t\right)-\gamma I_{h}\left(t\right)+\alpha I_{c}\left(t\right)\\ {\dot{R}}_{h}\left(t\right) & =\delta_{h}I_{h}\left(t\right)+\alpha R_{c}\left(t\right)-\gamma R_{h}\left(t\right), \end{array}$$

subject to the initial data:

(2) $$\begin{array}{ll} S_{c}\left(0\right) & =S_{c0},\;E_{c}\left(0\right)=E_{c0},\;I_{c}\left(0\right)=I_{c0},\;R_{c}\left(0\right)=R_{c0}\\ S_{h}\left(0\right) & =S_{h0},\;E_{h}\left(0\right)=E_{h0},\;I_{h}\left(0\right)=I_{h0},\;R_{h}\left(0\right)=R_{h0}. \end{array}$$

In the community, susceptible individuals *S*_*c*_(*t*) encounter infectious individuals both within the community *I*_*c*_(*t*) and the hub *I*_*h*_(*t*) through a force of infection Λ_*c*_(*t*) defined in (3), with transmission rate *β*_*c*_. As a result, they move to the compartment encountered *E*_*c*_(*t*), which represents individuals who have come into contact with sources of infection and are in the very early stage of exposure, but are not yet infectious in the classical epidemiological sense. Similarly, within the hub, susceptible individuals *S*_*h*_(*t*) encounter infectious individuals in both the community *I*_*c*_(*t*) and the hub *I*_*h*_(*t*), with a transmission rate *β*_*h*_ and a force of infection Λ_*h*_(*t*) defined in (3), passing to the encountered state *E*_*h*_(*t*) within the hub. Individuals in the encountered state in the community *E*_*c*_(*t*) become infectious at the rate *ɛ*_*c*_, moving to the infectious compartment *I*_*c*_(*t*), and subsequently recover at the rate *δ*_*c*_, transitioning to the recovered state *R*_*c*_(*t*). Similarly, individuals in the hub's encountered state *E*_*h*_(*t*) become infectious at rate *ɛ*_*h*_, then recover at rate *δ*_*h*_, moving to *I*_*h*_(*t*) and *R*_*h*_(*t*), respectively. Transitions between the hub and community occur for each compartment, where individuals in states *S*_*h*_(*t*), *E*_*h*_(*t*), *I*_*h*_(*t*), *R*_*h*_(*t*) within the hub move to corresponding states in the community at rate *γ*, while individuals enter the hub from the community at rate *α*, with both rates dependent on the respective compartment sizes *S*_*c*_(*t*), *E*_*c*_(*t*), *I*_*c*_(*t*), *R*_*c*_(*t*). Furthermore, susceptible individuals in the community *S*_*c*_(*t*) experience exposure at rate *p* when entering the hub, transitioning to the phase encountered in the hub at rate *αp*. Conversely, susceptible individuals in the hub *S*_*h*_(*t*) can be encountered at rate *p* when exiting the hub, transitioning to the encountered phase in the community at rate *γp*. The force of infection function for system (1) is defined as:

(3) $$\Lambda_{i}\left(t\right)=\frac{I_{c}\left(t\right)+I_{h}\left(t\right)}{1+k_{i}\left(I_{c}\left(t\right)+I_{h}\left(t\right)\right)},\;i=\left\{c,h\right\},$$

where the indices *c* and *h* denote the community and hub respectively, and $k_{i}\in R^{+}$ is the saturation parameter. It is notable that, although the incubation period of SARS-CoV-2, particularly the Omicron variant, which was the dominant strain in Canada in 2022, is generally reported to be approximately 2 to 4 days (Jansen, 2021; Ogata & Tanaka, 2023; Song et al., 2022), recent studies and outbreak data suggest that under certain conditions, particularly in high-density environments such as transportation hubs, transmission and onset of infectiousness can occur much more rapidly. This variability is influenced by factors such as host characteristics including age, immune status, and viral load at infection (Galmiche et al., 2023; Kong, 2020; Liu et al., 2021), which may substantially shorten incubation times. From a modeling perspective, including the transition rates *ɛ*_*c*_ and *ɛ*_*h*_ provides flexibility to represent both rapid and delayed progression from exposure to infectiousness. Even when focusing on short-term dynamics occurring over hours rather than days, these rates capture the biologically realistic, though very fast, progression without assuming it is instantaneous. Similarly, the recovery rates *δ*_*c*_ and *δ*_*h*_ remain small but nonzero to reflect gradual recovery processes. In the model diagrams, the transitions associated with *ɛ*_*c*_, *ɛ*_*h*_, *δ*_*c*_, and *δ*_*h*_ are represented by dashed lines to indicate that these rates are very small but not zero, highlighting their role in fast yet biologically meaningful transitions. The parameters of model (1) are listed in the following tables. Table 1 presents evidence-based parameters, while Table 2 contains the estimated parameters. In the following table, we present the infection relevant parameters for SARS-CoV-2, which was dominant in Ontario in November 2022.

### 4.2 Long-term dynamics

To capture the long-term behavior of COVID-19 transmission in the North York region beyond the short-term interactions between Finch Station and its surrounding community, we consider a classical SEIR framework incorporating vital dynamics and saturated incidence. The compartmental model is given by system (4), where the total population is divided into four epidemiological classes: susceptible individuals *S*(*t*), exposed individuals *E*(*t*), infectious individuals *I*(*t*), and recovered individuals *R*(*t*), visualized in Fig. 5 and is framed below:

(4) $$\left\{\begin{array}{l} \dot{S}=\beta -\Lambda \beta_{c}S-\mu S\\ \dot{E}=\Lambda \beta_{c}S-\left(\mu +\epsilon \right)E\\ \dot{I}=\epsilon E-\left(\mu +\nu +\delta \right)I\\ \dot{R}=\delta I-\mu R, \end{array}\right)$$

(5) $$S\left(0\right)=S0,\;E\left(0\right)=E_{0},\;I\left(0\right)=I_{0},\;R\left(0\right)=R_{0}.$$

Susceptible individuals are recruited into the population at the transmission birth rate *β* and are removed by natural and disease-induced mortality at rates *μ* and *ν*, respectively. They become exposed through effective contact with infectious individuals, governed by the effective community transmission rate *β*_*c*_ and the force of infection

(6) $$\Lambda =\frac{I}{1+kI},$$

which leads to new infections at rate Λ*S*(*t*). Here *k* > 0 is a saturation parameter representing the constant limitation of transmission when the number of infectious individuals becomes large. Exposed individuals progress to the infectious class at rate *ɛ*, infectious individuals recover at rate *δ*, and experience disease-induced mortality at rate *ν*, in addition to natural mortality. The inclusion of both natural and disease-related death terms enables realistic modeling of population turnover and long-term epidemic outcomes, including the possibility of disease persistence, endemic equilibrium, or eradication. The parameter values used in the model are summarized in Table 1. These parameters reflect epidemiological characteristics such as transmission, incubation, recovery, and mortality rates, with values informed by existing literature and public health data. By summing both sides of system 4 we obtain

(7) $$\dot{N}\left(t\right)=\beta -\nu I-\mu N\left(t\right),$$

where

(8) N(t)=S(t)+E(t)+I(t)+R(t).

Regarding the assumption of positive initial data and the positivity-preserving property of system (4), that is, the solution of system (4) remains positive whenever the initial data are positive, we conclude that

(9) $$N\left(t\right)\leq \frac{\beta }{\mu }+\left(N_{0}-\frac{\beta }{\mu }\right)e^{-\mu t}.$$

The above equation shows that, over the long term, the total number of individuals in the community approaches the constant value

(10) $$N\leq \frac{\beta }{\mu }.$$

Remark 4.1 In this study, we use a Holling type II functional form for the force of infection in both models 1 and 4. This formulation introduces nonlinear transmission dynamics, in which the infection rate saturates as the number of infected individuals becomes sufficiently large, a behavior that is consistent with real-world transmission processes.

## 5 Results

In this section, we analyze the outcomes of models (1) and (4) under various scenarios to examine their behaviors. By exploring different parameter values, we assess how changes in these parameters influence the models dynamics.

### 5.1 Short-term results

This section presents the results of model (1) for short-term dynamics under different scenarios. We focus on the number of individuals who have encountered the infection and entered the early stage of exposure, both in the community and within the hub. The analysis uses the evidence-based and estimated parameters listed in Table 1, Table 2 To quantify this, we calculate the number of encountered individuals in the hub and surrounding community over a single business day. Simulations are run for 21 h, corresponding to the subway station's operational hours from 5:00 a.m. to 2:00 a.m. the following day.

**Fig. 6:**
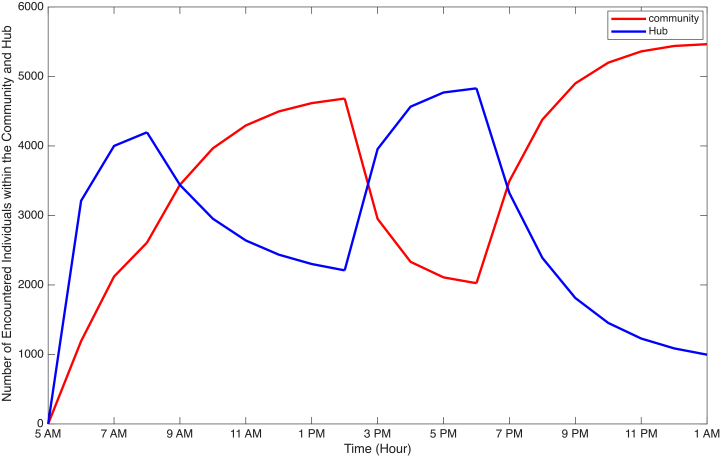
Number of encountered individuals in the community and within the hub over a single day.

Fig. 6 illustrates the dynamics of encountered individuals within the hub, alongside its surrounding community, which has an estimated total population of 7040. This analysis is based on the evidenced and estimated parameters outlined in Table 1, Table 2 The figure shows that the relative magnitude of encounters alternates between the hub and the community over the course of the day. During the early morning and morning commuting period (approximately 6–9 AM), the number of encountered individuals is higher in the hub than in the community. A similar dominance of the hub is observed again in the late afternoon (around 3–6 PM), corresponding to the evening commuting peak. In contrast, from late morning to early afternoon (roughly 10 a.m.–2 PM) and especially during the evening and late night hours (after about 7 p.m.), the number of encountered individuals is higher in the community than in the hub. In particular, the community clearly dominates in the late evening, when hub encounters steadily decline while community encounters continue to rise. Overall, the hub exhibits higher encounter levels primarily during peak commuting windows, whereas the community has higher encounter levels during off-peak daytime hours and most of the evening and night. This highlights a daily shift in where most interactions occur, from the hub during commute times to the community during non-commuting periods. This observed pattern underscores the crucial role of time and human mobility in shaping the dynamics of infectious diseases encounter within urban environments. The trends observed in the hub, as illustrated in this figure, align closely with the actual data on the passenger flow presented in Fig. 3, strengthening the relationship between the patterns of travelers and the levels of encounters. In summary, the hub exhibits a higher number of encountered individuals during peak commuting periods, reflecting intensified mobility associated with work-related and social activities. In contrast, during off-peak hours, the number of encountered individuals becomes higher in the community as individuals leave the hub and return to their residences, shifting interactions away from the hub.

**Fig. 7:**
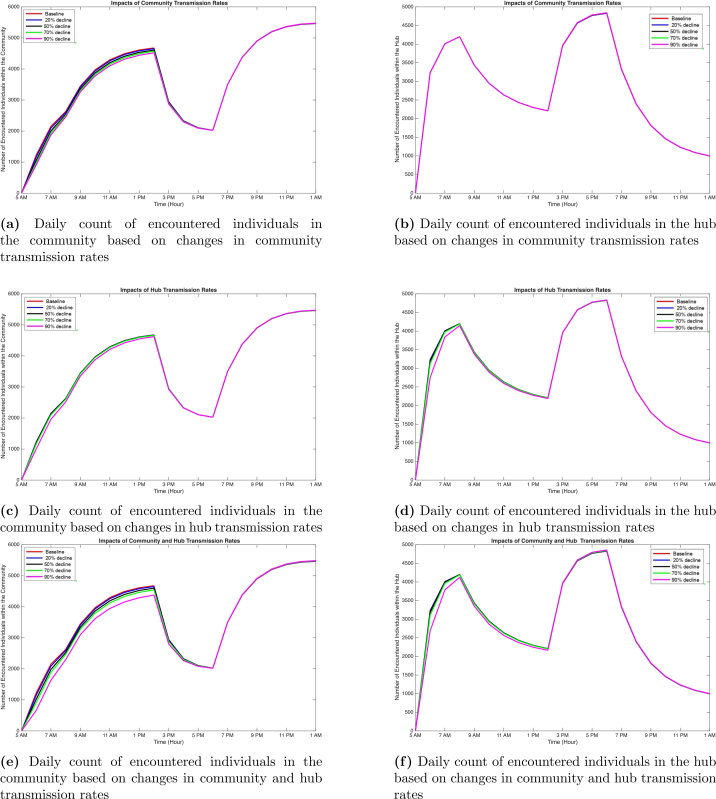
Impact of community and hub transmission rates on daily encountered individuals.

Fig. 7 illustrates how variations in transmission rates in both the community and the hub influence the number of encounters in these settings. Fig. 7a shows the effect of reducing the community transmission rate on the number of encountered individuals in the community over the course of a day. While the overall daily temporal pattern of encounters remains essentially unchanged across scenarios, any reduction in encounter numbers is small in magnitude and occurs primarily during the morning rush hours and the mid-day off-peak period. Quantitatively, a 20% reduction in the community transmission rate leads to only about a 0.48% decrease in the peak number of encountered individuals at 2 p.m. Similarly, a 50% reduction results in approximately a 1.44% decrease, a 70% reduction yields about a 2.28% decrease, and even a 90% reduction in transmission produces only around a 3.32% decrease in the peak number of encountered individuals at 2 p.m. Importantly, no noticeable changes are observed in the number of encountered individuals during the rest of the day, indicating that reductions in the transmission rate do not materially alter overall activity levels outside these specific time windows. This suggests that interventions affecting transmission efficiency mainly influence encounter counts during limited periods, while leaving the broader daily contact pattern essentially intact. Fig. 7b illustrates the effect of community transmission rates on the number of encountered commuters within the hub. Reducing the community transmission rate does not lead to any notable decrease in the number of encounters over the course of a single day. Fig. 7c shows the impact of the hub transmission rate on the number of encountered individuals in the community. Similar to the community transmission case, the effects of changes in the hub transmission rate are only noticeable during the early morning rush hours and the mid-day off-peak period. Specifically, a 20% and 50% reductions in the hub transmission rate results in a 0.03% and 0.11% decreases, respectively in the peak number of encountered individuals in the community at 2 p.m. Likewise, 70% and 90% reductions lead to approximately 0.27% and 1.16% decreases in the community encounter peak, respectively. No discernible changes are observed during the rest of the day. Fig. 7d visualizes the impact of hub transmission rates on the number of encountered commuters within the hub over a single day. As in the previous cases, the effects are most evident during the morning rush hours and the mid-day off-peak period. The results show that a 20%, 50%, 70%, and 90% reductions in the hub transmission rate causes a 0.02%,10%, 025%, and 1.24% decreases, respectively in the peak number of encountered commuters during the first peak, corresponding to the morning rush hour at 8 a.m. These effects diminish during the mid-day off-peak period, with declines of approximately 0.015%, 0.06%, 0.16%, and 0.84% for 20%, 50%, 70%, and 90% reductions in the hub transmission rate, respectively, at the minimum point occurring at 2 p.m. The impact becomes negligible during the rest of the day. Overall, the results indicate that the impacts of both community and hub transmission rates are only slightly significant during the morning rush hours and the mid-day off-peak period, with no noticeable changes in the number of encountered individuals during the rest of the day. Notably, changes in community transmission rates have a more pronounced effect on the number of encountered individuals in the community than do changes in hub transmission rates. In contrast, variations in the hub transmission rates have a stronger impact on the number of encountered commuters within the hub than do changes in community transmission rates. This indicates that, in the short-term dynamics, reductions in transmission rates do not produce substantial changes in encounter patterns. Fig. 7e illustrates the impact of simultaneous changes in both community and hub transmission rates on the number of encountered individuals in the community. Consistent with Fig. 7a and b, the most pronounced effects occur during the morning rush hours and midday off-peak periods. The results show that a 20% reduction in both transmission rates leads to a 0.52% decrease in the number of encountered individuals at 2 p.m. Correspondingly, reductions of 50%, 70%, and 90% yield decreases of 1.64%, 2.86%, and 6.39%, respectively, at the same time point. Fig. 7f follows a pattern similar to Fig. 7d, with minor differences. Specifically, simultaneous reductions of 20%, 50%, 70%, and 90% in both community and hub transmission rates result in decreases of 0.016%, 0.10%, 0.30%, and 1.83%, respectively, in the number of encountered individuals within hubs at 8 a.m. A comparison across Fig. 7a–f indicates that simultaneous reductions in transmission rates are more effective in decreasing the number of encountered individuals in the community than in the hubs. In contrast, the impact on reducing encounters within hubs remains relatively limited.

**Fig. 8:**
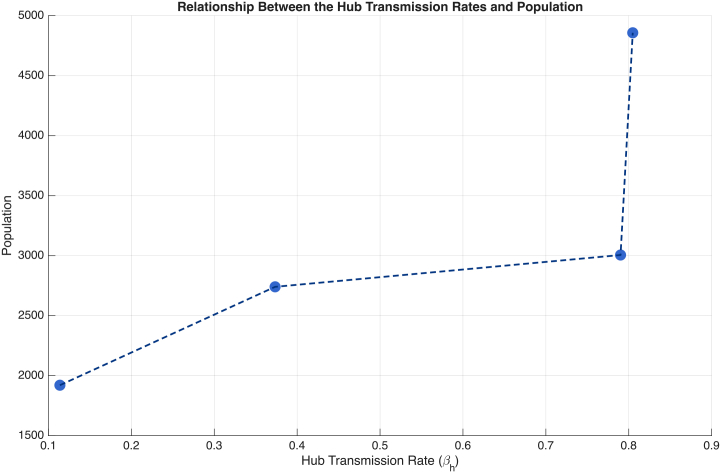
Parameter dependency between the hub transmission rate and the number of commuters visiting the hub during a single day.

Fig. 8 illustrates the relationship between the hub transmission rate and the average total number of commuters visiting the hub per hour across different time intervals of the day. The results show that this relationship is not strictly linear and varies across time periods. In general, higher population levels tend to be associated with higher transmission rates, but notable deviations from a simple monotonic pattern are observed. For instance, during the morning rush hours, the average hourly population in the hub is approximately 3005, corresponding to an estimated transmission rate of 0.8048. During the morning off-peak period, the average population decreases to about 2740, and the transmission rate drops substantially to 0.1138. However, this trend is not uniform throughout the day. In the afternoon rush hours, despite a much larger average population of approximately 4857, the transmission rate is 0.7901, which is slightly lower than the morning rush value. Similarly, during the evening off-peak period, the population decreases to about 1920, yet the transmission rate remains relatively high at 0.3734. These results indicate that while crowd size influences the transmission rate, the relationship is modulated by time-of-day effects and is not solely determined by the number of individuals present in the hub.

**Fig. 9:**
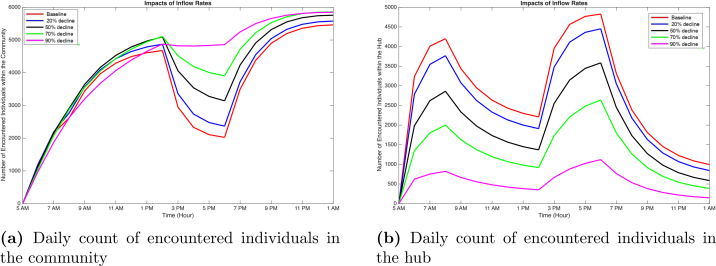
Impact of different inflow rates on daily encountered individuals.

Fig. 9 illustrates the effect of varying inflow rates from the community to the hub on the number of encountered individuals in both environments. As the inflow rate decreases, fewer individuals enter the hub, which reduces the number of encountered individuals there, while the number of encounters in the community increases due to the accumulation of individuals who remain outside the hub. Fig. 9a and b therefore display opposite trends. For the community (Fig. 9a), changes in the inflow rate affect the number of encountered individuals differently across time intervals. During the morning rush hours and the mid-day off-peak period, when the peak occurs at 2 p.m., reductions in the inflow rate lead to higher numbers of encounters. Specifically, 20% and 90% reductions in the inflow rate result in approximately a 4.19% increase in the peak number of encounters, while a 50% reduction leads to an 8.91% increase and a 70% reduction produces about a 9.19% increase. In contrast, during the afternoon rush hours, the number of encountered individuals decreases and reaches a minimum at 6 p.m. In this interval, the minimum number of encounters increases by 16.99% for a 20% reduction in the inflow rate, by 55.20% for a 50% reduction, and by 92.86% and 140.04% for 70% and 90% reductions, respectively. The number of encountered individuals then rises during the evening off-peak hours and reaches its maximum at the end of the operating period (1 a.m.). At this time, the peak increases by 2.11% for a 20% reduction in the inflow rate, by 5.39% for a 50% reduction, and by nearly 7% for both 70% and 90% reductions. Overall, these results indicate that the largest relative increases in the number of encountered individuals in the community occur during the afternoon rush hours. Within the hub (Fig. 9b), the effects are far more pronounced than in the community. During the morning rush hours, the first peak at 8 a.m. decreases by approximately 10%, 31%, 52%, and 80% in response to 20%, 50%, 70%, and 90% reductions in the inflow rate, respectively. A similar pattern is observed during the mid-day off-peak period. By 2 p.m., the number of encountered commuters decreases by about 13%, 37%, 58%, and 84% for the same sequence of inflow reductions. Overall, the rates of decline during the morning rush hours and the mid-day off-peak period are nearly identical. During the afternoon rush hours, the second peak also declines, by approximately 7%, 25%, 45%, and 76%, again corresponding to 20%, 50%, 70%, and 90% reductions in the inflow rate. This trend persists into the evening off-peak hours, where the minimum number of encountered commuters at 1 a.m. decreases by about 15%, 40%, 60%, and 85% for the same respective reductions in the inflow rate. The close correspondence between reductions in inflow and decreases in encounter peaks within the hub underscores the importance of controlling population movement during outbreak scenarios. Mitigation measures such as travel restrictions or reduced transit capacity may therefore be effective strategies for limiting disease spread. Moreover, the results suggest that even modest reductions in the inflow rate can yield proportional decreases in outbreak intensity. The close correspondence between reductions in inflow and decreases in encounter peaks within the hub underscores the importance of controlling population movement during outbreak scenarios. Mitigation measures such as travel restrictions or reduced transit capacity may therefore be effective strategies for limiting disease spread. Moreover, the results suggest that even modest reductions in the inflow rate can yield proportional decreases in encounter intensity within the hub. These findings highlight the interdependent nature of disease dynamics between communities and transit hubs, where individual mobility between these environments shapes encounter patterns. Specifically, decreasing the inflow rate alleviates congestion within the hub, reducing crowd density during rush periods and thereby lowering the number of encounters in the hub. At the same time, fewer individuals entering the hub implies that more individuals remain in the community, which can increase the number of encounters there. Consequently, inflow control primarily reduces hub encounters and may indirectly reduce hub transmission, *β*_h_, by limiting close contacts in crowded settings.

$$\text{inflow rate}↓\;\;\Rightarrow \;\;\text{encounters}↓\;\;\Rightarrow \;\;\beta_{\text{h}}↓$$

**Fig. 10:**
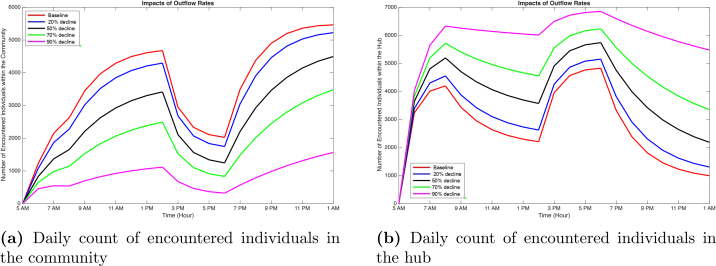
Impact of different outflow rates on daily encountered individuals.

Fig. 10 demonstrates the influence of varying outflow rate from the hub to the community on the number of encountered individuals in both areas. A reduction in outflow rate decreases the number of individuals exiting the hub, causing the community components of model (1) to become less crowded while the hub components become more crowded. In Fig. 10a, variations in the outflow rate have a substantial impact on the number of encountered individuals in the community across different periods of the operating day. During the morning rush hours and the mid-day off-peak period, when the peak occurs at 2 p.m., a 20% reduction in the outflow rate decreases the number of encountered individuals by approximately 8% at the peak. Larger reductions in the outflow rate of 50%, 70%, and 90% lead to decreases of about 27%, 47%, and 76% at the peak, respectively. During the afternoon rush hours, when the number of encountered individuals follows a decreasing trend and reaches a minimum at 6 p.m., the minimum number of encounters is reduced by approximately 14%, 39%, 59%, and 84% for 20%, 50%, 70%, and 90% reductions in the outflow rate, respectively. Conversely, during the evening off-peak hours, the number of encountered individuals increases. In this period, the number of encounters decreases by about 4%, 18%, 36%, and 71% at the peak when the outflow rate is reduced by 20%, 50%, 70%, and 90%, respectively. Fig. 10b illustrates the opposite effect in the hub. A 20% reduction in the outflow rate leads to an 8% increase in the peak number of encountered commuters during the morning rush hours, which occur at 8 a.m. As the outflow rate is further reduced by 50%, 70%, and 90%, the peak number of encountered commuters increases by approximately 24%, 36%, and 51%, respectively. During the mid-day off-peak hours, the number of encountered commuters in the hub decreases and reaches its minimum at 2 p.m. At this time, the number of encountered commuters increases by about 19%, 62%, 106%, and 172% when the outflow rate is reduced by 20%, 50%, 70%, and 90%, respectively. During the afternoon rush hours, when the number of encountered commuters increases and reaches its peak at 6 p.m., the peak value rises by approximately 7%, 19%, 29%, and 42% for 20%, 50%, 70%, and 90% reductions in the outflow rate, respectively. Finally, during the evening off-peak hours, the number of encountered commuters decreases and reaches its minimum at 1 a.m. the following day. At this time, the number of encountered commuters in the hub increases by approximately 31%, 119%, 236%, and 450% when the outflow rate is reduced by 20%, 50%, 70%, and 90%, respectively. When comparing Fig. 9, Fig. 10, it becomes clear that inflow and outflow rates have opposite impacts on outbreak dynamics within the community and hub. Reducing inflow rate leads to fewer encountered individuals, while reducing outflow rate increases the number of encounters within the hub and, consequently, affects the surrounding community. Interestingly, while outflow rate reductions reduce the peak in the community, they result in a significant rise in encounter within the hub, particularly during rush hours. The results show that decreasing outflow rate increases traffic congestion in the hub, which consequently raises the number of encountered commuters in the hub and indirectly increases the effective transmission rate within the hub. Since more individuals remain in the hub and do not return to the community, the number of encountered individuals in the community decreases, leading to an indirect reduction in the effective hub transmission rate:

$$\begin{array}{ll} \text{outflow rate}↓ & \;\;\Rightarrow \;\;\text{hub congestion}↑\\ & \;\;\Rightarrow \;\;\text{hub encounters}↑\\ & \;\;\Rightarrow \;\;\beta_{\text{h}}↑ \end{array}$$

**Fig. 11:**
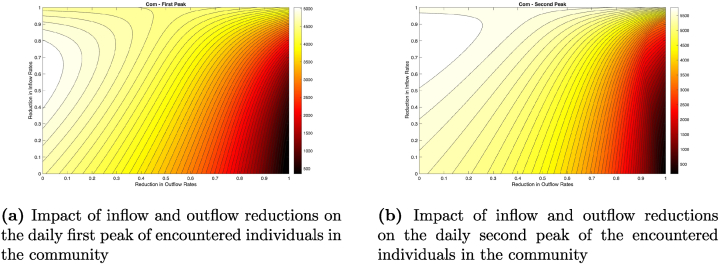
Impact of inflow and outflow reductions on the daily peaks of the encountered individuals in the hub.

**Fig. 12:**
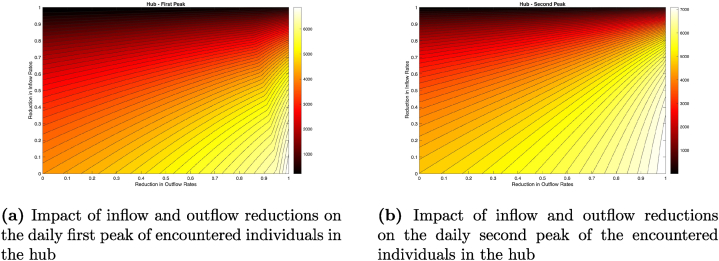
Impact of inflow and outflow reductions on the daily peak of the encountered individuals in the hub.

Fig. 11 illustrates the effects of varying inflow and outflow rates on the first and second daily peaks in the number of encountered individuals in the community, which occur at 2 p.m. and 1 a.m., respectively. Figs. 11a and b further show that reducing the outflow rate while keeping the inflow rate constant decreases both peaks, since fewer individuals leave the hub and, consequently, fewer commuters enter the community. In contrast, reducing the inflow rate while holding the outflow rate constant increases both peaks because more individuals remain in the community, leading to a higher number of encounters. This demonstrates the sensitivity of the system to changes in traffic flow within the hub. The impact of either inflow and outflow rates individually investigates in Fig. 9, Fig. 10. When both inflow and outflow rates are reduced simultaneously, the system exhibits a more nuanced response. A coupled 20% reduction in both rates results in decreases of 3.6% in the first peak and 1.9% in the second peak. More substantial changes are observed for larger reductions. When both rates are reduced by 70%, the first and second peaks decrease by 23% and 18.71%, respectively. The effects become even more pronounced for a 90% reduction. A coupled 90% reduction in both inflow and outflow rates yields decreases of 24.2% and 26.6% in the first and second peaks, respectively. Overall, these results highlight the dominant role of the outflow rate in shaping encounter peaks in the community. Reducing the inflow rate tends to increase both peaks by retaining more individuals in the community, whereas reducing the outflow rate decreases the peaks by limiting the number of individuals entering the community. Simultaneous reductions in both rates generally lower both peaks, confirming that the outflow rate has the stronger influence on community crowding.Fig. 12 presents the influence of adjusting inflow and outflow rates on the first and second daily peaks in the number of encountered individuals in the hub. Figs. 12a and b further show that when the outflow rate is reduced while the inflow rate remains constant, both daily peaks increase. This occurs because fewer individuals leave the hub, resulting in a buildup of people and consequently higher numbers of encountered individuals. In contrast, when the inflow rate is reduced while the outflow rate stays constant, both peaks decline because fewer individuals are entering the hub, leading to a reduced population of encountered individuals. This interplay between inflow and outflow rates highlights the sensitivity of the system to changes in traffic flow within the hub. The impact of either inflow and outflow rates individually investigates in Fig. 9, Fig. 10. The combined effect of reducing both inflow and outflow rates produces a more complex pattern. When both inflow and outflow rates decrease, it means that the rate of individuals entering either the community and hub decreases. For instance,a coupled 20% reduction in both inflow and outflow rates leads to a 2% decrease in the first peak and a 0.75% increase in the second peak. More substantial changes occur with larger reductions. A coupled 70% reduction produces a 25.8% decrease in the first peak and a 12% decrease in the second. The effects become even more pronounced with a 90% reduction. A coupled 90% reduction in both inflow and outflow rates yields a 57.8% decrease in the first peak and a 41.1% decrease in the second peak. These findings highlight the strong influence of inflow and outflow rates on encounter peaks within the hub. Reducing the inflow rate decreases both daily peaks by limiting the number of individuals entering the hub, whereas reducing the outflow rate increases the peaks by causing individuals to remain inside, leading to higher crowding. Simultaneous reductions in both rates produce mixed effects, generally lowering both peaks in the hub, confirming the role of inflow rate in the hub crowding. This underscores the importance of managing population movement within the hub to control crowding and reduce transmission risk.

### 5.2 Long-term results

This section analyzes how the daily number of individuals encountered in the community during short-term dynamics influences the number of infected individuals in the community over the subsequent three months.

**Fig. 13:**
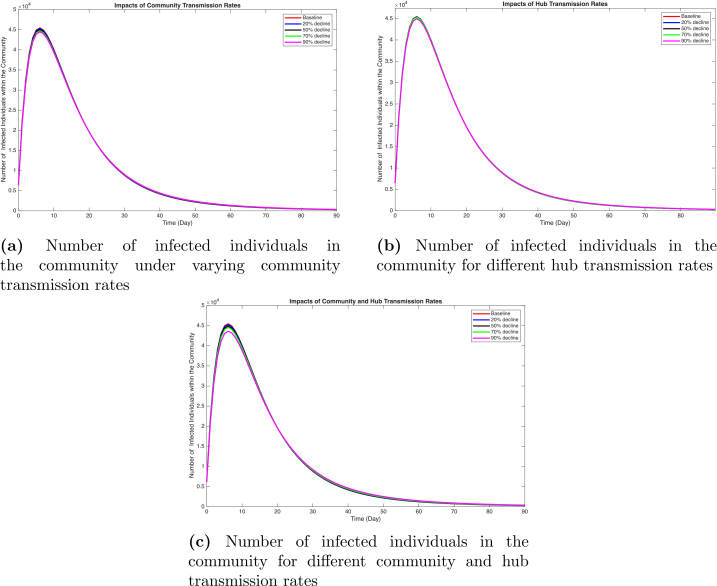
Impact of community and hub transmission rates on the number of infected individuals over a 90-day period.

Fig. 13 illustrates how community and hub transmission rates affect the daily number of encountered individuals in the community in the short-term dynamics and, consequently, the number of infected individuals over the long-term period of 90 days. Fig. 13a specifically shows the impact of community transmission rates on the number of infected individuals in the community. The results indicate that infections increase during the first 10 days, reach a peak around day 10, and then gradually decline, approaching a plateau toward the end of the three-month period. Overall, reductions in community transmission during a single business day in the short-term dynamics have only a minor effect on the number of infected individuals over the subsequent 90 days. For example, a 20% reduction in the community transmission rate leads to only a 0.387% decrease in the peak number of infected individuals. Larger reductions of 50%, 70%, and 90% result in approximate decreases of 1.06%, 1.58%, and 2.18%, respectively, with the peak occurring around day 10 in all cases. Fig. 13b shows the effect of reducing the hub transmission rate during a single business day on long-term infection dynamics in the community. The results indicate even smaller impacts, with reductions of approximately 0.03%, 0.12%, 0.28%, and 1.11% in the number of infected individuals for 20%, 50%, 70%, and 90% decreases in the hub transmission rate, respectively. Overall, these findings suggest that changes in either community or hub transmission rates during a single day have only a very limited influence on long-term infection dynamics. A comparison between Fig. 7, Fig. 13 further confirms that, while these transmission rates can affect the number of encountered individuals in the short-term dynamics, their impact on the long-term number of infected individuals remains minimal. Fig. 13c illustrates the impact of simultaneously varying community and hub transmission rates on the number of infected individuals in the community in the long term. As shown, the overall pattern is similar to that observed in Fig. 13a, with slight differences. Specifically, simultaneous reductions of 20%, 50%, 70%, and 90% in both community and hub transmission rates over a single day result in decreases of 0.42%, 1.06%, 1.99%, and 4.04%, respectively, in the number of infected individuals. A comparison between Figs. 13a and c indicates that reducing both community and hub transmission rates simultaneously is more effective than reducing each one individually in lowering the number of infected individuals in the long term.

**Fig. 14:**
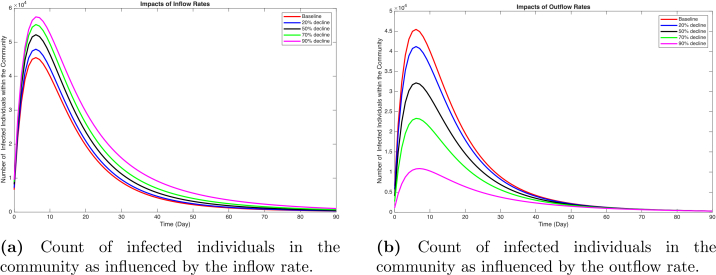
Impact of mobility rates on the number of infected individuals over a 90-day period.

Fig. 14 showcases the impact of mobility rates, including inflow and outflow rates, on the number of encountered individuals in the community during the short-term dynamics and how this influences the number of infected individuals in the community over the subsequent three months. Fig. 14a visualizes the impact of reductions in inflow rates during a single business day and their effects on the number of infected individuals in the community over a 90-day period. The results indicate that a 20% reduction in the inflow rate during a single business day leads to a 5.48% increase in the number of infected individuals in the community over the 90-day period. Further reductions of 50%, 70%, and 90% in the inflow rate result in increase of approximately 14.82%, 21.40%, and 26.38%, respectively. These results are related to the effect of inflow rates on the number of encountered individuals in the community during the short-term dynamics, as depicted in Fig. 9a. Fig. 14b illustrates the impact of the outflow rate on the number of encountered individuals in the community during the short-term dynamics and its effect on the number of infected individuals in the community over the subsequent three months. The findings show that reductions of 20%, 50%, 70%, and 90% in the outflow rate during the short-term dynamics lead to decreases of approximately 9.39%, 29.27%, 48.69%, and 76.27% in the number of infected individuals in the community, respectively. These results are related to the effect of outflow rates on the number of encountered individuals in the community during a single day in the short-term dynamics, as visualized in Fig. 10a. As it is seen in Fig. 9, Fig. 10a, overall, the long-term outcomes are similar to the trends observed in the short-term dynamics.

**Fig. 15:**
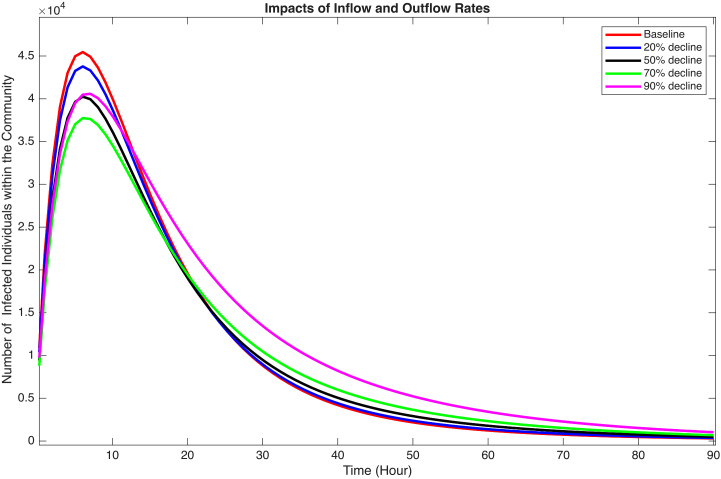
Effects of simultaneous reductions in mobility rates on infections over a 90-Day Period.

Fig. 15 illustrates the impact of simultaneously reducing the inflow and outflow mobility rates on the cumulative number of infected individuals over a three-month period. The results show that joint reductions of 20%, 50%, and 70% in both inflow and outflow rates lead to progressively larger decreases in the number of infectees, specifically by approximately 3.69%, 10.96%, and 16.96%, respectively. This monotonic trend does not persist for the most extreme scenario. When both rates are reduced by 90%, the total number of infectees decreases by only about 10.73%, which is smaller than the reduction observed under the 70% scenario. This non-monotonic response indicates that the relationship between mobility reduction and long-term infection burden is nonlinear. Such behavior can arise from the structure of the model and the interaction between mobility and transmission dynamics, where further reductions in movement may yield diminishing or saturated effects on cumulative infections. In addition, this pattern may reflect sensitivity to parameter interactions and the underlying data used in the simulations. Overall, these results suggest that while moderate to strong simultaneous reductions in inflow and outflow are effective in reducing infections, increasingly severe restrictions do not necessarily translate into proportionally larger long-term benefits within this modeling framework.

## 6 Discussion

This study provides a comprehensive examination of the mechanisms driving the spread of respiratory infectious diseases, such as COVID-19, within an urban transit hub and its surrounding community. Using Finch Subway Station in North York, Canada, as a case study, we analyzed short-term and long-term transmission dynamics through deterministic ordinary differential equation (ODE) models. Our results indicate that mobility-related factors, particularly inflow and outflow rates, play a more important role in mitigating infection transmission than transmission rates themselves, in both short-term and long-term dynamics. Specifically, reducing the inflow rate decreases encounters in the hub by easing congestion but increases encounters in the community, whereas reducing the outflow rate increases crowding and encounters in the hub while decreasing encounters in the community, thereby indirectly elevating the risk of transmission in the hub. Moreover, simultaneous reductions in inflow and outflow rates decrease the number of encountered individuals in both the community and the hub. In the long-term dynamics, reducing either the community or hub transmission rate has only a limited effect on lowering the peak number of infections. It is noteworthy that simultaneous reductions in community and hub transmission rates are more effective than reducing either transmission rate alone, in both short-term and long-term dynamics. By contrast, decreasing the inflow rate during a single business day slightly increases long-term community infections, while reducing the outflow rate leads to a notable decrease in infections, consistent with the short-term trends. Simultaneous reductions in inflow and outflow rates reduce the number of infected individuals in the community. Overall, jointly reducing inflow and outflow rates emerges as the most effective strategy for mitigating the number of encountered individuals in the community and in the short term and the number of infected individuals in the community in the long term. Several limitations must be considered when interpreting the results. First, precise population data for the surrounding community are unavailable, necessitating parameter estimation using the least squares method. Additionally, the assumption of constant total population over a single day simplifies modeling but may not fully capture real-world dynamics. From a policy perspective, effective management of infection risk requires coordinated interventions that reduce community transmission rate, which lowers encounters both in the community and hubs, while hub-specific measures primarily control local crowding. Regulating inflow and outflow at hubs can alleviate congestion and indirectly influence transmission rates, with reduced inflow rate lowering encounters and reduced outflow rate increasing hub crowding but decreasing community exposure. Long-term protection is best achieved through sustained community-level measures that flatten infection peaks, while hub-targeted strategies serve as complementary controls. Overall, integrating transmission control with mobility management is essential for minimizing both short- and long-term infection risks In summary, reducing community transmission rate lowers encounters and peak infections across both the community and hub, while hub-targeted measures mainly limit local crowding, and mobility patterns further influence exposure, highlighting the need to integrate community-level control with hub management to minimize short- and long-term infection risks. Policymakers can leverage these insights to implement targeted, practical interventions that balance public health safety with urban mobility needs, thereby minimizing disease transmission while maintaining essential transit operations.

## CRediT authorship contribution statement

**Rahele Mosleh:** Writing – review & editing, Writing – original draft, Visualization, Validation, Software, Resources, Project administration, Methodology, Investigation, Formal analysis, Data curation, Conceptualization. **Mina Shafadeh:** Writing – review & editing, Writing – original draft, Project administration, Methodology, Formal analysis, Data curation, Conceptualization. **Bushra Majeed:** Writing – review & editing, Writing – original draft, Project administration, Methodology, Conceptualization. **Julien Arino:** Supervision, Methodology. **Abbas Ghasemi:** Supervision, Methodology, Investigation, Conceptualization. **Edward W. Thommes:** Supervision. **Ali Asgary:** Supervision. **Herman Hui:** Data curation. **Jianhong Wu:** Supervision, Funding acquisition.

## Declaration of interest statements

Rahele Mosleh declares no conflicts of interest. Jianhong Wu has received research funding from the NSERC–Sanofi Industrial Research Chair program, *Vaccine Mathematics, Modelling and Manufacturing*, and from the Ontario Research Fund–Research Excellence program, *Integrating AI with Disease Transmission Dynamics Models for Informed Prevention and Control of Outbreaks in Indoor and Mass Gathering Settings*, which supports the Mathematics for the Laboratory of Industrial and Applied Mathematics (LIAM) initiative. Edward W. Thommes is an employee of Sanofi and may hold shares and/or stock options in the company. The remaining authors declare no conflicts of interest. Jianhong Wu serves as Editor-in-Chief of the *Journal of Infectious Disease Modelling*, and Julien Arino and Edward W. Thommes serve as members of the Editorial Board of the same journal.

## Figures and Tables

**Fig. 16:**
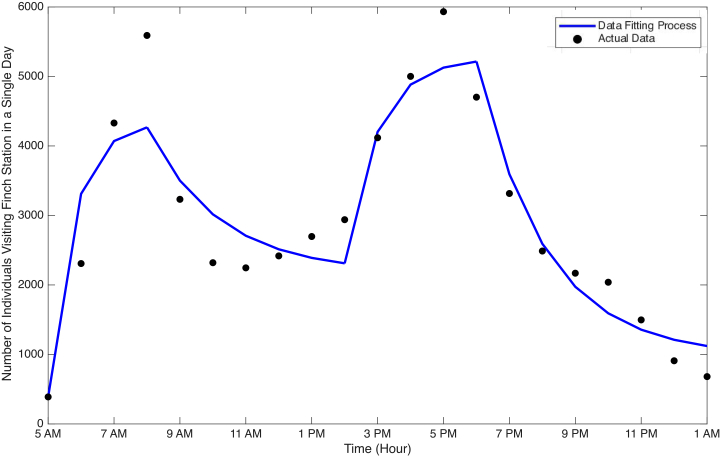
Data fitting process at Finch Subway Station and its surrounding community: over a single business day in November 2022.

## Appendices

### A Data Fitting Process for the Deterministic Disease Model

We apply the least squares method to estimate the model parameters, as presented in Table 2. To fine-tune these parameters, we use the *fminsearch* function from the MATLAB Optimization Toolbox (Inc, 2023). For each parameter, we gather relevant data and calculate a 95% confidence interval by averaging the data and determining the standard deviation. The confidence intervals were then derived assuming a normal distribution. During this process, we ensure that the confidence intervals encompass the estimated parameters. Adjusting the intervals to more accurately capture the estimated parameter values. The data fitting process depicted in Fig. 16, based on the real data visualized in Fig. 3, specifically highlights the total ridership trends at Finch Subway Station. In summary, this process involves estimating and refining model parameters, ensuring the results are statistically reliable, and visually comparing the model's predictions with observed data to assess the accuracy of the fit.

Fig. 16 illustrates the data-fitting procedure for the model (1)–(2) using the least-squares method. To remain consistent with the empirical ridership patterns in Fig. 3, we divide the day into four time intervals morning rush hours, mid-day off-peak hours, afternoon rush hours, and evening off-peak hours and estimate a separate set of parameters for each. This segmentation enables the model to capture daily variations in human mobility and contact patterns in the transit system, resulting in a more accurate and reliable representation of the observed dynamics.
